# Inclusion of microbe-derived antioxidant during pregnancy and lactation attenuates high-fat diet-induced hepatic oxidative stress, lipid disorders, and NLRP3 inflammasome in mother rats and offspring

**DOI:** 10.29219/fnr.v63.3504

**Published:** 2019-08-23

**Authors:** Zhen Luo, Xue Xu, Sen Zhao, Takami Sho, Wenli Luo, Jing Zhang, Weina Xu, Kong Hon, Jianxiong Xu

**Affiliations:** 1 Shanghai Key Laboratory of Veterinary Biotechnology, School of Agriculture and Biology, Shanghai Jiao Tong University, Shanghai, China; 2 Shanghai Vocational College of Agriculture and Forestry, Shanghai, China; 3 Shanghai Chuangbo Biotechnology Institute, Shanghai, China

**Keywords:** high-fat diet, liver, microbe-derived antioxidant, NLRP3 inflammasome, rats

## Abstract

**Objective:**

This study aimed to evaluate the effects of microbe-derived antioxidant (MA) on high-fat diet (HFD)-induced hepatic lipid disorders in mother rats and offspring.

**Methods:**

A total of 36 female rats were randomly divided into three groups at the beginning of pregnancy: the control group (CG), HFD, and HFD with 2% MA. Mother rats were slaughtered at the first and 10th day of lactation (L1 and L10) and offspring were slaughtered at L10. The plasma and liver of mother rats, and liver of offspring were collected.

**Results:**

The results showed that MA reversed HFD-induced activities of inducible nitric oxide synthase (iNOS) and antioxidative enzymes in liver of mother rats and offspring. In addition, MA reduced HFD-induced lipid accumulation through decreasing the low-density lipoprotein cholesterol (LDLC) content in plasma of mother rats and improving hepatic fatty acid synthase (FAS) in mother rats and offspring. MA decreased HFD-induced hepatic alkaline phosphatase (AKP) activity in liver of mother rats and offspring. Furthermore, MA reduced HFD-activated nucleotide-binding oligomerization domain-like receptor containing pyrin domain 3 (NLRP3) inflammasome in liver of mother rats and offspring.

**Conclusions:**

MA supplementation reversed HFD-induced hepatic oxidative stress, lipid accumulation, NLRP3 inflammasome, and function in mother rats and offspring, suggesting MA can be functional ingredients to improve maternal-fetal health.

## Popular scientific summary

- MA reversed HFD-induced activities of iNOS and antioxidative enzymes in liver of mother rats and offspring.
- MA reduced HFD-induced lipid accumulation through decreasing the LDLC content in plasma of mother rats and improving hepatic FAS in mother rats and offspring.
- MA reduced HFD-activated NLRP3 inflammasome in liver of mother rats and offspring.

Epidemiological studies have indicated that obesity-related metabolic diseases are major risk for public health. Maternal overnutrition during pregnancy and lactation predisposes offspring metabolism (1, 2). Animal studies recently have shown that high-fat diet (HFD) during pregnancy and lactation increases hepatic oxidative stress, lipid accumulation, inflammatory cytokines, and activation of signaling pathways, predisposing offspring to the development of metabolic disorders such as insulin resistance and nonalcoholic fatty liver disease (3–5). Nucleotide-binding oligomerization domain-like receptor containing pyrin domain 3 (NLRP3) is a central regulator of innate immunity in response to endogenous molecules. Once activated, NLRP3 inflammasome increases the caspase-1 expression, which can promote maturation of IL-1β and IL-18 (6). NLRP3 inflammasome participates in immune dysfunction leading to chronic inflammation, insulin resistance, and metabolic disorders in HFD-induced obesity in mice (7, 8). Furthermore, maternal obesity was reported to increase hepatic natural killer cells and TNFα in prenatal offspring (9). Whether HFD activated inflammatory response through NLRP3 inflammasome both in liver of mother rats and offspring during pregnancy and lactation has not been investigated.

Bioactive compounds and plant extracts are reported to have protective and beneficial effects against diet-induced metabolic disorders (10). Furthermore, antioxidant blend interacted in a complex network and showed synergistic action *in vitro* and *in vivo*, which is promising in functional foods (11, 12). Microbe-derived antioxidants (MA) are a new-type compounds fermented by probiotics, containing isoflavones, superoxide dismutase (SOD), glutathione, Se, and vitamin C and E, which showed superior free radical scavenging capacity *in vitro* (13). To date, it is uncertain whether MA supplementation during gestation and lactation has beneficial synergies to improve HFD-induced hepatic metabolic disorders in rats and offspring. Thus, in the present study, we hypothesized that dietary MA supplementation during pregnancy and lactation would attenuate HFD-induced hepatic redox status and lipid accumulation through NLRP3 inflammasome in mother rats and offspring. The hypothesis was explored by investigating the hepatic redox status, hepatic function, lipid metabolism, and NLRP3 inflammasome in mother rats and offspring.

## Materials and methods

### Animals and diets

The experiment was conducted according to the guidelines of Shanghai Jiao Tong University Institutional Animal Care and Use Committee. The MA is made from the fruits of Sea buckthorn and Rosa roxburghii, which were fermented by beneficial bacteria such as *Bacillus subtilis*, *Lactobacillus*, Beer yeast through solid–liquid complex fermentation and processing methods, such as extraction, concentration, inactivation, and freeze-drying. MA (KB-120, dry powder) was provided by Shanghai Jiang Han Biotechnology and the main components of MA are 503 mg/kg Fe, 367 mg/kg Mn, 1.07 mg/kg Cu, 0.18 mg/kg Se, 194,000 U/100 g SOD, 322 mg/100 g vitamin C, 908 μg/100 g vitamin E, 4.43% total isoflavones, 1.37% isoflavones, 886 mg/100 g glutathione, 82.4 mg/100 g total saponins, 4.21% total amino acids, and 0.146% taurine (13). A total of 36 nonpregnancy Sprague-Dawley (SD) female rats (body weight 250–270 g) were fed with a standard diet for 3 days to adapt to the environment. Then they were mixed and mated with 12 male SD rats (three females and one male in a cage). A vaginal plug is considered as successful pregnancy. Then each pregnant rat was transferred to a single cage (25 ×40 cm) and fed separately. They were randomly allocated into three groups (12 rats per group) fed with the following diets: standard diet (CG), 80% standard diet + 20% lard (HFD), and 78% standard diet + 20% lard + 2% MA (HFDA). Rats were free access to drinking water and food in a controlled environment (23 ± 2°C, 50–60% relative humidity, 12-h light and 12-h darkness cycles). The experiment began at the first day of pregnancy and ended at the 10th day of lactation (L10). Six mother rats from each group were given pentobarbital sodium (45 mg/kg, P3761; Sigma, USA) through intraperitoneal injection according to the body weight and slaughtered at the first day of lactation (L1) and L10. The plasma samples were centrifuged at 5,000 × *g* for 20 min; the supernatants and liver samples were stored at −80°C. Offspring from each litter (*n* = 6) were also given pentobarbital sodium and slaughtered at L10.The liver tissues were sampled and immediately frozen at −80°C.

### Determination of oxidative stress parameters in liver

The liver samples were weighed and homogenized in saline solution (1:9, w/v), and the supernatants were collected after centrifuging at 10,000 × *g* for 10 min. Protein concentrations were measured according to the instructions of the bicinchoninic acid (BCA) protein assay kit (P0010; Beyotime Biotech, Shanghai, China). The concentrations of H_2_O_2_, malondialdehyde (MDA) and total antioxidant capacity (T-AOC), the activities of total and inducible nitric oxide synthase (tNOS and iNOS), SOD, glutathione peroxidase (GSH-Px), catalase (CAT), and xanthine oxidase (XOD) in the liver were determined by measuring the absorbance at 405, 532, 520, 530, 530, 550, 412, 405, 530 nm according to the protocols of respective commercial kits (Nanjing Jiancheng Bioengineering Institute, Nanjing, China).

### Determination of lipid profiles in plasma and liver

The contents of triglyceride (TG), total cholesterol (TC), high-density lipoprotein cholesterol (HDLC) and low-density lipoprotein cholesterol (LDLC), oxidized low-density lipoprotein (Ox-LDL) in plasma, and TG in liver were determined by a colorimetric assay, and the absorbance was measured at 500, 510, 546, 546, 450, and 500 nm, following the commercial kits (Nanjing Jiancheng Bioengineering Institute).

### Enzyme-linked immunosorbent assay (ELISA)

The activities of caspase-3, caspase-8, caspase-9, and fatty acid synthase (FAS) in liver, and the contents of Ox-LDL, IL-1β, IL-18, and caspase-1 in plasma were determined according to the protocols of the respective ELISA kits (Nanjing Jiancheng Bioengineering Institute and Shanghai Yili Biological Technology). Briefly, the plates were coated with their corresponding antibodies followed by detection with a horseradish peroxidase-labeled substrate after incubation at 37°C for 10 min. The absorbance was recorded in a microplate reader (Synergy 2; BioTek, USA) at 450 nm. A four parameter logistic curve-fit was generated using ELISA Calc software v0.1 (Comple-Software, Iowa City, IA). The concentrations of samples were calculated from the standard curve.

### Determination of hepatic function

The activities of alanine aminotransferase (ALT), aspartate aminotransferase (AST), and alkaline phosphatase (AKP) in plasma and liver tissues were determined by measuring the absorbance at 510, 510, 520 nm according to the commercial kits (Nanjing Jiancheng Bioengineering Institute).

### RNA isolation, cDNA synthesis and real-time PCR

The liver samples were homogenized in liquid nitrogen, then lysed in 700 μL TRK Lysis Buffer (R6841-01; Omega, USA) containing 2-mercaptoethanol (0482; Amresco, USA). RNA was extracted according to the protocol of the E.Z.N.A. total RNA kit I (R6841-01; Omega). The concentrations of RNA were quantified using a NanoDrop Lite Spectrophotometer (Thermo Fisher Scientific, USA). Then 2,000 ng of RNA were reverse transcribed into cDNA using PrimeScript™ RT reagent Kit (RR047A; Takara, Japan). The real-time PCR reaction was performed using a SYBR Green premix Ex Taq (RR420A; Takara) with Applied Biosystems 7500 Real-Time PCR system (Thermo Fisher Scientific). Amplification conditions were initially 95°C for 30 sec, 40 cycles of 95°C for 5 sec, and 60°C for 34 sec, with a stepwise increase in the temperature from 60 to 95°C to obtain the melting curve data. The primer sequences were designed according to previous studies, as shown in Table 1. Results were expressed as a fold of the CG, using the 2^-(ΔΔ^*^Ct^*^)^, where ΔΔ*Ct* = (*Ct*_Target_ *– Ct*_GAPDH_)_treatment_ – (*Ct*_Target_ – *Ct*_GAPDH_)_control_.

**Table 1:** The primer sequences used in this study

Name	Forward (5′–3′)	Reverse (5′–3′)	References
GAPDH	CCCTTCATTGACCTCAACTAC	CTTCTCCATGGTGGTGAAGAC	40
IL-1β	TTCTTTGAGGCTGACAGACC	CGTCTTTCATCACACAGGAC	40
IL-18	GTGAACCCCAGACCAGACTG	CCTGGAACACGTTTCTGAAAGA	41
NLRP3	AGTGGATAGGTTTGCTGGGATA	CTGGGTGTAGCGTCTGTTGAG	40
Caspase-1	AGTGTAGGGACAATAAATGG	GATGGACCTGACTGAAGC	40
Caspase-3	GAGACAGACAGTGGAACTGACGATG	GGCGCAAAGTGACTGGATGA	42
Caspase-8	GCGACAGGTTACAGCTCTCC	GCAGCCTCTGAAATAGCACC	43
Caspase-9	CTGGCCCAGTGTGAATACCT	CTCAGTCAACTCCTGGGCTC	43
FAS	GGAACTGCTTTCTCTTTCTGC	AACGCTCCTCTTCAACTCCA	44
CPT1α	ATCCACCATTCCACTCTGCT	TGTGCCTGCTGTCCTTGATA	44
PPAR-α	CCATACAGGAGAGCAGGGATT	CCACCATTTCAGTAGCAGGA	44
PPAR-γ	CTGACCCAATGGTTGCTGATTAC	CCTGTTGTAGAGTTGGGTTTTTTCA	44
ACC	ATTCTGGCGGATCAGTATGG	AGCAATAGCAGCAGGAGCTT	44
MACD	GTCGCGCCAGACTACGATAA	GCCAAGACCACCACAACTCT	45
PGC1α	ATGAATGCAGCGGTCTTAGC	TGGTCAGATACTTGAGAAGC	46

### Statistical analysis

Data were presented as mean ± standard deviation (SD). The data were analyzed with one-way analysis of variance (ANOVA) followed by least significant difference (LSD) test, using the statistical software SPSS 20.0 (SPSS, Chicago, IL, USA). *p* < 0.05 was considered statistically different.

## Results

### Oxidative stress parameters in liver

Oxidative stress parameters in the liver of mother rats are shown in Table 2. At L1, MA supplementation significantly lowered the HFD-induced activities of tNOS, iNOS, and MDA content, but did not improve (*p* > 0.05) HFD-induced activities of CAT, GSH-Px, SOD, and XOD and T-AOC content in the liver of mother rats. At L10, MA significantly decreased the HFD-induced the activities of tNOS and SOD and contents of H_2_O_2_ and MDA (*p <* 0.05), but had no effects on HFD-induced activities of iNOS, CAT, XOD, and GSH-Px and the content of T-AOC (*p >* 0.05).

**Table 2:** Oxidative stress parameters in the liver of mother rats at L1, L10, and offspring

Item	CG	HFD	HFDA
Mother rats, L1			
tNOS (U/g total protein)	401.42 ± 50.51^b^	477.73 ± 14.25^a^	412.07 ± 58.33^b^
iNOS (U/g total protein )	207.16 ± 16.18^b^	290.16 ± 50.93^a^	232.28 ± 28.42^b^
H_2_O_2_ (mmol/g total protein)	7.76 ± 1.59	8.78 ± 2.10	7.78 ± 1.92
MDA (nmol/mg total protein)	0.54 ± 0.11^ab^	0.62 ± 0.08^a^	0.46 ± 0.09^b^
CAT (U/mg total protein)	41.37 ± 7.92^a^	24.66 ± 10.30^b^	30.35 ± 12.13^ab^
GSH-Px (U/mg total protein)	906.00 ± 34.40^a^	788.26 ± 69.39^b^	861.59 ± 19.53^b^
SOD (U/mg total protein)	366.56 ± 15.59^a^	314.52 ± 18.39^b^	318.31 ± 17.88^b^
T-AOC (U/mg total protein)	1.98 ± 0.11^a^	1.57 ± 0.14^b^	1.55 ± 0.06^b^
XOD (U/g total protein)	16.85 ± 0.65^a^	13.00 ± 1.49^b^	13.02 ± 1.09^b^
Mother rats, L10			
tNOS (U/g total protein)	487.99 ± 54.80^b^	641.64 ± 81.55^a^	526.21 ± 66.86^b^
iNOS (U/g total protein)	202.15 ± 14.03^b^	284.42 ± 2.66^a^	262.37±11.27^a^
H_2_O_2_ (mmol/g total protein)	9.29 ± 2.09^ab^	11.55 ± 4.78^a^	7.32 ± 2.01^b^
MDA (nmol/mg total protein)	0.65 ± 0.11^ab^	0.88 ± 0.10^a^	0.52 ± 0.21^b^
CAT (U/mg total protein)	60.52 ± 8.81^a^	40.58 ± 4.60^b^	41.82 ± 6.11^b^
GSH-Px (U/mg total protein)	876.92 ± 91.79^a^	653.64 ± 37.05^b^	681.53 ± 41.69^b^
SOD (U/mg total protein)	379.12 ± 40.01^b^	532.38 ± 59.06^a^	331.76 ± 80.40^b^
T-AOC (U/mg total protein)	2.34 ± 0.36^a^	1.48 ± 0.25^b^	1.68 ± 0.23^b^
XOD (U/g total protein)	19.46 ± 2.42^a^	14.97 ± 0.76^b^	14.26 ± 1.17^b^
Offspring			
tNOS (U/g total protein)	696.32 ± 95.05^b^	1163.98 ± 185.77^a^	984.39 ± 31.14^b^
iNOS (U/g total protein)	473.67 ± 2.43^b^	510.98 ± 5.69^a^	483.79 ± 19.06^b^
H_2_O_2_ (mmol/g total protein)	8.00 ± 0.69	8.44 ± 1.17	7.43 ± 1.28
MDA (nmol/mg total protein)	0.77 ± 0.12^b^	1.20 ± 0.15^a^	1.05 ± 0.15^a^
CAT (U/mg total protein)	34.10 ± 2.90^b^	37.89 ± 2.87^a^	30.59 ± 1.49^b^
GSH-Px (U/mg total protein)	253.45 ± 42.40^b^	309.36 ± 55.94^a^	304.93 ± 33.63^ab^
SOD (U/mg total protein)	273.87 ± 10.29^a^	279.68 ± 6.33^a^	217.59 ± 5.12^b^
T-AOC (U/mg total protein)	1.04 ± 0.10^a^	0.80 ± 0.03^b^	0.86 ± 0.04^b^
XOD (U/g total protein)	11.12 ± 0.93^b^	12.53± 1.06^a^	11.15 ± 0.35^b^

Data were presented as mean ± standard deviation (SD). The superscript letters a and b represent significant differences (*p* < 0.05) among treatments.

H_2_O_2_, hydrogen peroxide; MDA, malondialdehyde; tNOS, total nitric oxide synthetase; iNOS, inducible nitric oxide synthase; CAT, catalase; GSH-Px, glutathione peroxidase; SOD, superoxide dismutase; T-AOC, total antioxidant capacity; XOD, xanthine oxidase; CG, control group; HFD, high-fat diet; HFDA, HFD + 2% microbe-derived antioxidant (*n* = 6).

Oxidative stress parameters in the liver of offspring are shown in Table 2. MA significantly decreased (*p <* 0.05) the HFD-induced activities of tNOS, iNOS, CAT, SOD, and XOD, but had no effects on HFD-induced activity of GSH-Px, contents of MDA and T-AOC (*p >* 0.05).

### Lipid profiles in the plasma of mother rats

At L1 (Table 3), MA significantly decreased the HFD-induced content of LDLC (*p <* 0.05) in plasma of mother rats, but had no effects on the HFD-induced content of TC (*p >* 0.05). The contents of TG, HDLC, and Ox-LDL were not affected by treatments (*p >* 0.05). At L10, MA significantly decreased HFD-induced contents of TC, HDLC, and LDLC (*p <* 0.05). The contents of TG and Ox-LDL were not affected by treatments (*p >* 0.05).

**Table 3:** The contents of lipid profiles in plasma of mother rats

Item	CG	HFD	HFDA
Mother rats, L1			
TG (mmol/L)	1.22 ± 0.06	1.44 ± 0.36	1.27 ± 0.22
TC (mmol/L)	1.73 ± 0.13^a^	2.33 ± 0.24^b^	2.34 ± 0.06^b^
HDLC (mmol/L)	1.75 ± 0.08	1.57 ± 0.30	1.47 ± 0.24
LDLC (mmol/L)	0.22 ± 0.02^a^	0.35 ± 0.03^b^	0.25 ± 0.08^a^
Ox-LDL (μg/L)	33.21 ± 1.53	35.80 ± 2.92	33.95 ± 1.50
Mother rats, L10			
TG (mmol/L)	0.88 ± 0.14	1.04 ± 0.12	0.93 ± 0.15
TC (mmol/L)	1.89 ± 0.24^a^	3.38 ± 0.47^b^	1.95 ± 0.39^a^
HDLC (mmol/L)	1.38 ± 0.16^a^	1.05 ± 0.14^b^	1.38 ± 0.32^a^
LDLC (mmol/L)	0.23 ± 0.03^a^	0.38 ± 0.06^b^	0.24 ± 0.02^a^
Ox-LDL (μg/L)	28.33 ± 3.23	27.18 ± 1.82	28.12 ± 3.11

Data were presented as mean ± standard deviation (SD). Values with different letters differ significantly (*p* < 0.05).

TG, triglyceride; TC, total cholesterol; HDLC, high-density lipoprotein cholesterol; LDLC, low-density lipoprotein cholesterol; Ox-LDL, Oxidized low-density lipoprotein; CG, control group; HFD, high-fat diet; HFDA, HFD + 2% microbe-derived antioxidant (*n* = 6).

### Lipid metabolism in liver

**Fig. 1:**
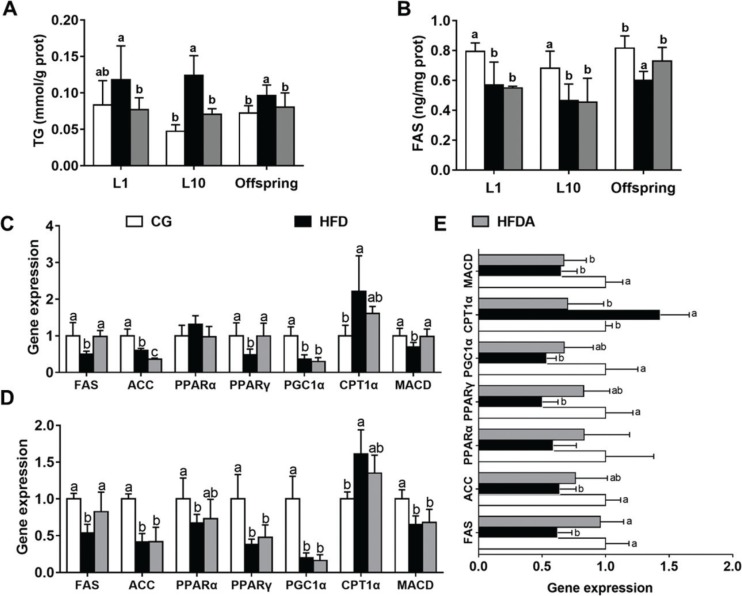
Lipid profiles TG (A), FAS (B) in liver of mother rats at L1 and L10. Gene expression of lipid metabolism in the liver of mother rats at L1 (C), L10 (D), and offspring (E). Values with different letters differ significantly (*p* < 0.05). TG, triglyceride; FAS, fatty acid synthase; ACC, acetyl-CoA carboxylase; PPARα, peroxisome proliferator-activated receptor alpha; PPARγ, peroxisome proliferator-activated receptor gamma; PGC1α, peroxisome proliferator-activated receptor gamma coactivator 1-alpha; CPT1α, carnitine palmitoyltransferase-1α; MACD, medium-chain acyl-CoA dehydrogenase; CG, control group; HFD, high-fat diet; HFDA, HFD +2% microbe-derived antioxidant (*n* = 6).

Lipid metabolism parameters in liver of mother rats and offspring are shown in Fig. 1A–C. At L1 and L10 (Fig. 1A and B), MA significantly decreased (*p <* 0.05) the HFD-induced content of TG, but had no effect on the HFD-induced activity of FAS (*p >* 0.05). In the offspring, MA significantly recovered HFD-induced (*p <* 0.05) content of TG and activity of FAS.

The gene expression of lipid metabolism in liver is presented in Fig. 1D–F. At L1, MA significantly reversed (*p <* 0.05) HFD-induced gene expression of FAS, peroxisome proliferator-activated receptor gamma (PPARγ), and medium-chain acyl-CoA dehydrogenase (MACD), but did not reverse (*p >* 0.05) gene expression of acetyl-CoA carboxylase (ACC), peroxisome proliferator-activated receptor gamma coactivator 1-alpha (PGC1α), and carnitine palmitoyltransferase-1α (CPT1α) in the liver of mother rats. At L10, MA significantly increased (*p <* 0.05) HFD-induced gene expression of FAS, but did not recover the HFD-induced expression of other genes (*p >* 0.05). In the offspring, MA significantly reversed (*p <* 0.05) HFD-induced gene expression of FAS and CPT1α, but did not reverse (*p >* 0.05) HFD-induced gene expression of ACC, PPARγ, PGC1α, and MACD in the liver.

### The activities of AST, ALT, and AKP in plasma and liver

**Fig. 2:**
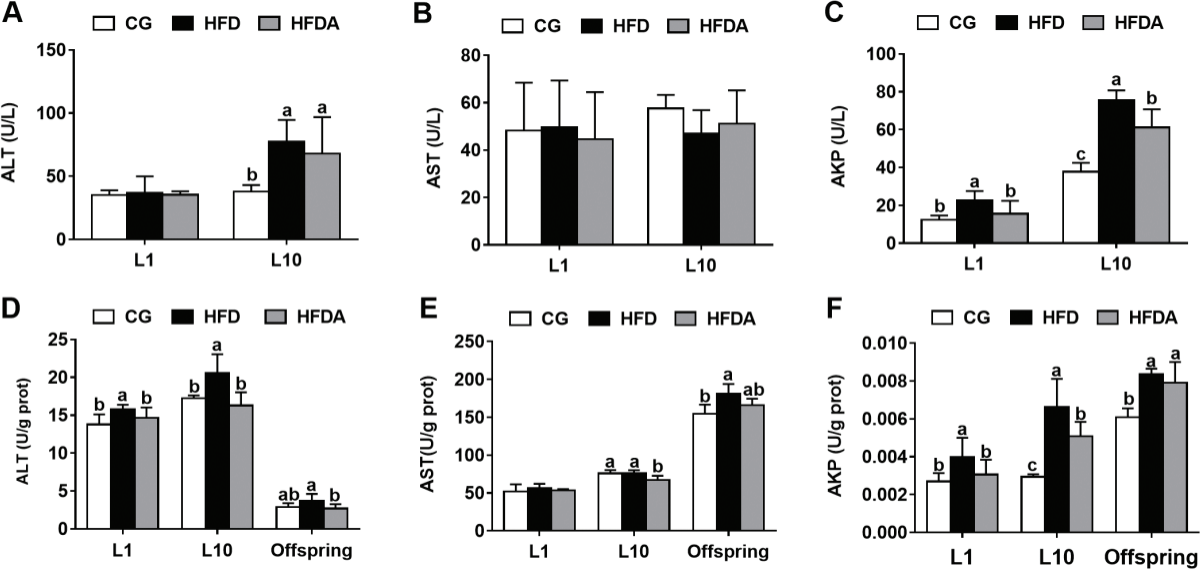
The activities of ALT (A), AST (B), and AKP (C) in plasma of mother rats at L1 and L10. The activities of ALT (D), AST (E), and AKP (F) in the liver of mother rats at L1, L10, and offspring. Values with different letters differ significantly (*p* < 0.05). AST, aspartate aminotransferase; ALT, alanine aminotransferase; AKP, alkaline phosphatase; CG, control group; HFD, high-fat diet; HFDA, HFD +2% microbe-derived antioxidant (*n* = 6).

The activities of AST, ALT, and AKP in plasma of mother rats are shown in Fig. 2A–C. At L1 and L10, MA significantly (*p <* 0.05) decreased the HFD-induced activity of AKP, but did not affect the activities of AST and ALT (*p >* 0.05).

The activities of AST, ALT, and AKP in liver of mother rats are shown in Fig. 2D–F. At L1, MA significantly decreased (*p <* 0.05) HFD-induced activities of ALT and AKP, but did not affect the activity of AST (*p >* 0.05). At L10, MA significantly decreased (*p <* 0.05) the activities of ALT, AKP, and AST compared with the HFD (*p >* 0.05). In the offspring, MA significantly decreased the activity of ALT (*p <* 0.05), but did not affect HFD-induced activities of AST and AKP (*p >* 0.05).

### The activities of caspases and gene expression in liver

**Fig. 3:**
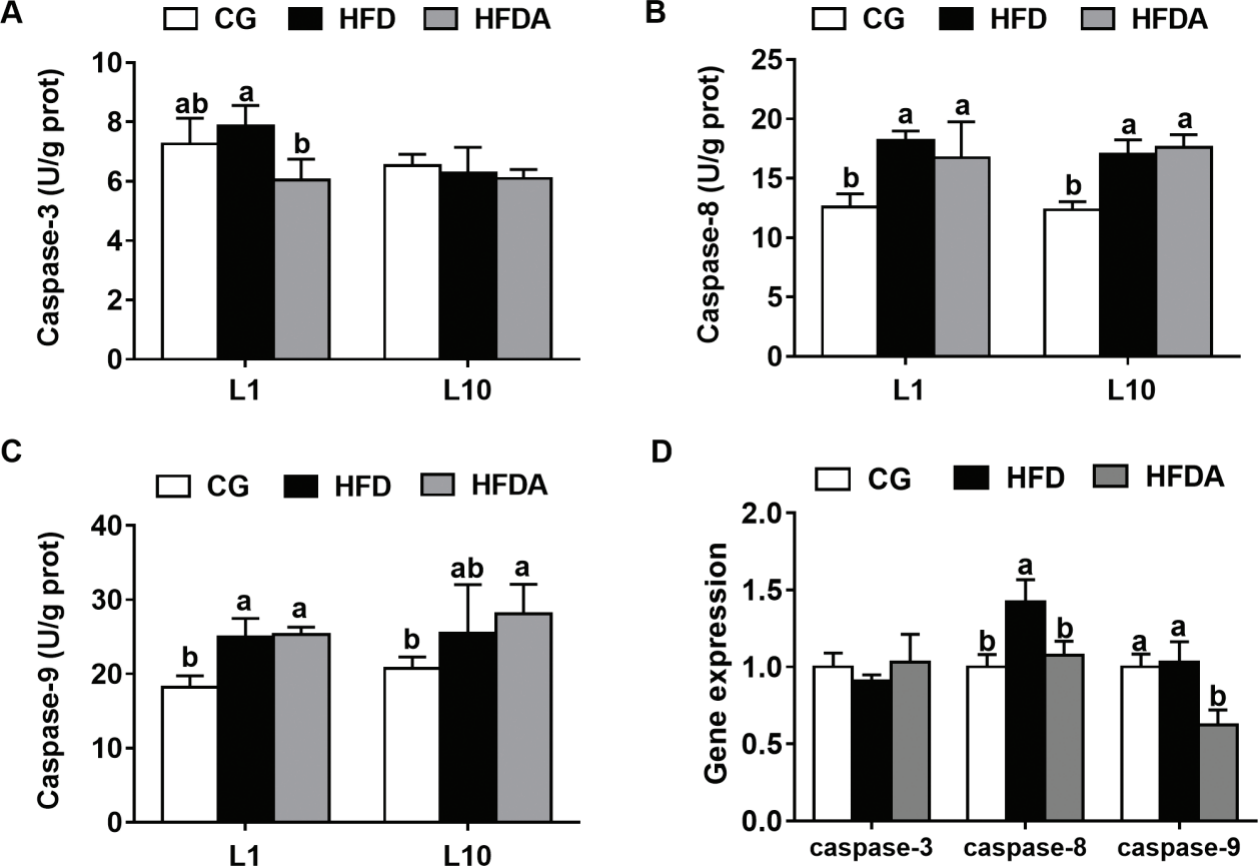
The activities of caspase-3 (A), caspase-8 (B), and caspase-9 (C) in liver of mother rats at L1 and L10. Gene expression of caspase-3, caspase-8, and caspase-9 in offspring (D). Values with different letters differ significantly (*p* < 0.05). CG, control group; HFD, high-fat diet; HFDA, HFD +2% microbe-derived antioxidant (*n* = 6).

The activities of caspases in liver of mother rats are shown in Fig. 3A–C. At L1, MA significantly decreased (*p* < 0.05) the activity of caspase-3, but had no effects on HFD-induced activities of caspase-8 and caspase-9 (*p >* 0.05). At L10, MA had no effect on the activities of caspase-3, caspase-8, and caspase-9 (*p >* 0.05).

The gene expression of caspases in offspring is presented in Fig. 1D. MA significantly decreased (*p <* 0.05) the HFD-induced gene expression of caspase-8 and caspase-9, but had no effects on the gene expression of caspase-3 (*p >* 0.05).

### NLRP3 inflammasome in plasma and liver

**Fig. 4:**
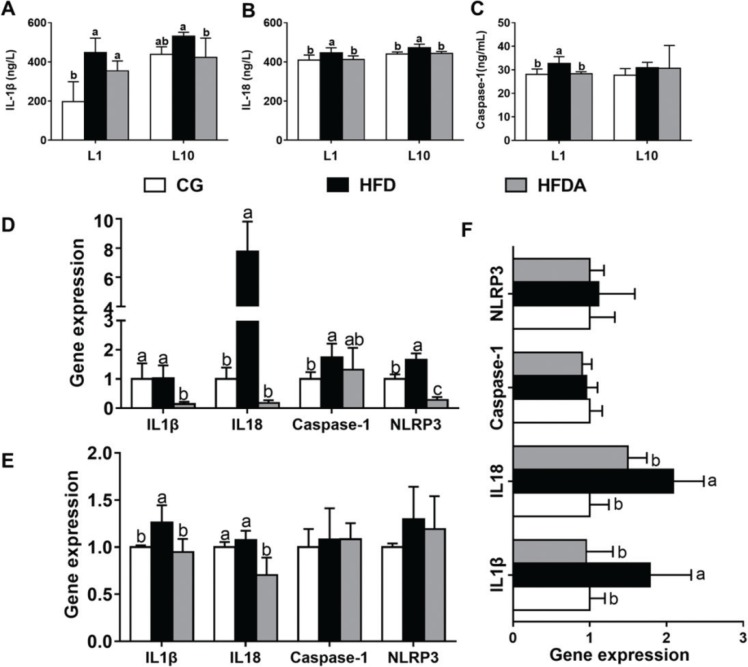
The activities of IL-1β (A), IL-18 (B), and caspase-1 (C) in plasma of mother rats at L1 and L10. Gene expression of NLRP3 inflammasome pathway in liver of mother rats at L1 (D), L10 (E), and offspring (F). Values with different letters differ significantly (*p* < 0.05). CG, control group; HFD, high-fat diet; HFDA, HFD +2% microbe-derived antioxidant (*n* = 6).

The contents of IL-1β, IL-18, and caspase-1 in plasma of mother rats at L1 and L10 are presented in Fig. 4A–C. At L1, MA significantly reversed (*p <* 0.05) HFD-induced contents of IL-1β, IL-18, and caspase-1 in plasma of mother rats. At L10, the contents of IL-1β and IL-18 decreased (*p <* 0.05) in the HFDA compared with the HFD. The content of caspase-1 was not affected by dietary treatments.

Gene expression of NLRP3 inflammasome in liver of mother rats and offspring is shown in Fig. 4D–F. At L1, MA significantly decreased (*p <* 0.05) the HFD-induced gene expression of NLRP3, IL-1β, and IL-18, but had no effect on caspase-1 expression (*p* > 0.05). At L10, MA significantly decreased (*p <* 0.05) HFD-induced gene expression of IL-1β and L-18, but did not affect the gene expression of NLRP3 and caspase-1 (*p* > 0.05) in the offspring.

## Discussion

Our previous study has suggested that MA supplementation decreased HFD-induced body weight and feed intake of mother rats, which could affect the individual birth weight of offspring (14). Furthermore, MA decreased the liver weight of mother rats compared with CG and HFD at L1, but had no effect at L10 (Supplemental Table 1). From a health standpoint, the present study showed that MA supplementation during pregnancy and lactation improved HFD-induced hepatic redox status and function, decreased LDLC content in plasma and FAS activity in liver, and inhibited NLRP3 inflammasome in mother rats and offspring, implying that MA is beneficial and promising to prevent chronic diseases or metabolic syndrome in offspring.

The liver is a major site of fatty acid and TG synthesis and regulator of whole lipid metabolism homeostasis. Hepatic lipid disorders are often associated with obesity and metabolic syndromes (15). Hepatic fat accumulation has been suggested to be a result of the increase of free fatty acids uptake and *de novo* hepatic lipogenesis, decrease of fatty acids oxidation, and alteration in lipoprotein delivery (16). LDLC is responsible for delivering TG and cholesterol to skeletal muscle and adipose tissue for energy expenditure and storage, while HDLC facilitates translocation of cholesterol from peripheral tissue to liver (17). ACC and FAS are mainly involved in *de novo* lipid synthesis. CPT1α, PPARs, PGC1α, and MACD are responsible for fatty acid β-oxidation and esterification (5, 18). In the present study, MA normalized HFD-induced content of LDLC in plasma and TG in liver at L1 and L10, TC and HDLC contents at L10 in mother rats, improved the gene expression of FAS in mother rats and offspring, and attenuated the content of TG and the activity of FAS in offspring, suggesting that MA decreased HFD-induced hepatic lipid accumulation in mother rats and offspring probably through decreasing hepatic lipogenesis and altering lipoprotein transport during gestation and lactation. Previous studies have reported that supplementation with probiotics and probiotic-fermented soymilk effectively decreased lipid profiles through inhibiting lipogenic gene expression and increasing lipolytic gene expression (19, 20). The different mechanisms of regulating lipid metabolism possibly depend on the ingredients after probiotic fermentation. Of note, it is reported that bile salt hydrolase produced by probiotic fermentation is mainly responsible for hydrolyzing conjugated bile salts and reducing the serum cholesterol content (21, 22). Whether bile salt hydrolase exists in MA is unknown and needs to be verified.

iNOS is the enzyme to produce NO (nitrogen radical), which is a key mediator of immune and inflammatory responses (23). XOD produces O_2_^−^ and H_2_O_2_ (oxygen radicals) in liver (24). The increase of FFA intake or lipid overload has been reported to induce an increase of reactive stress species (ROS) production (25). In this study, HFD increased the activities of tNOS, iNOS, and XOD, but MA decreased the activities of tNOS and iNOS in liver of both mother rats and offspring, suggesting MA is mainly responsible for scavenging NO and inflammation *in vivo*. These results are consistent with the findings from our previous studies showing that MA inhibited DPPH (a marker of nitrogen radical) *in vitro* and LPS-induced hepatic NO content in rats (13, 26). SOD, GSH-Px, and CAT play an important role in preventing O_2_^−^ and H_2_O_2_-induced oxidative damage (27). MDA is one of the biomarker of lipid peroxidation. MA supplementation did not change the activity of CAT and GSH-Px, but decreased the content of MDA in liver of mother rats, while MA increased the activity of CAT, but did not change the MDA content in liver of offspring, indicating that MA probably plays a different role in preventing HFD-induced hepatic oxidative stress in mother rats and offspring under different physical conditions. Similarly, a recent study reported that resveratrol intake during pregnancy and lactation is effective under context-specific metabolic stress or depends on the cellular redox status (28). On the contrary, the synergistic effects of multiple antioxidants probably have significant advantages. Combination of vitamin C and meso-2,3-dimercaptosuccinic acid showed a better efficacy than monotherapy arsenic–fluoride coexposure in rats (29). Thus, these results indicated that antioxidant blend MA was considered as a promising functional food in preventing chronic diseases. Of note, HFD increased the activity of SOD in liver of mother rats at L10 and offspring, suggesting a compensatory response in resisting oxidative stress (30).

The apoptosis response is regulated by either the death receptor pathway or the mitochondrial pathway in cells, depending on different stress sources. The death receptors can activate caspase-8 and downstream caspase-3, while the other pathway induces release of mitochondria protein cytochrome c to activate caspase-9 and downstream caspase-3 (31). In the present study, results showed that MA did not recover HFD-induced activities of caspase-8 and caspase-9 in liver of mother rats, but decreased gene expression of caspase-8 and caspase-9 in offspring, suggesting that MA could effectively attenuate maternal HFD-induced hepatic apoptosis in offspring and protect fetus from maternal factors. The excessive apoptosis of hepatocytes could result in hepatic dysfunction. AST and ALT are intracellular enzymes in the liver, which are specific indicators for hepatocellular injury when released into blood circulation. The increase of AKP in blood is considered as hepatic bile duct obstruction (32). Previous studies have reported that HFD supplementation during pregnancy and lactation affected hepatic function and metabolism in offspring (33, 34). Dietary supplementation with *Lactobacillus plantarum* KCTC3928 increased bile acid secretion and lowered cholesterol content in C57BL/6 mice (22). Furthermore, genistein supplementation in laying broiler breeder hens increased cholesterol 7α-hydroxylase (CYP7A1) expression, an enzyme responsible for conversion of cholesterol into bile acids, in the liver of offspring (35). The present results showed that MA decreased HFD-induced activity of AKP in plasma of mother rat, and ALT and AKP in liver of mother rats and offspring. These results suggested that dietary probiotics or antioxidants effectively recovered hepatic function through decreasing HFD-induced hepatocellular injury and clearing hepatobiliary system both in mother rats and offspring. Thus, the promotion of bile acid secretion and metabolism in offspring by dietary MA supplementation will be explored in our future studies.

NLRP3 inflammasomes are large protein complexes that represent the first line of the innate immunity. Recent study have reported that HFD-induced NLRP3 inflammasome participated in inflammation and metabolic disorders (7). Furthermore, saturated fatty acid palmitate activated NLRP3 inflammasome in bone marrow-derived macrophages through AMPK-autophagy-ROS pathways, while this regulating pathway is unique in contrast to conventional NLRP3 agonists such as ATP and nigericin, suggesting the complex regulation of NLRP3 inflammasome in response to fatty acids (8, 36). In fact, NLRP3 activation needs a two-step process: the first signal is to activate NF-κB pathway and transcriptional expression of inflammasome, and the second signal is to induce inflammasome formation and instigate caspase-1 activation (37). The present study showed that MA inhibited HFD-induced NLRP3 inflammasome in liver of mother rats at L1 and decreased the gene expression of IL-1β and IL-18 in mother rats at L10 and offspring, but did not affect the gene expression of NLRP3 and caspase-1 in mother rats at L10 and offspring. The specific mechanism of differences in HFD-activation of NLRP3 inflammasome between mother rats and offspring still needs further studies. Dietary antioxidants such as red raspberries, quercetin, and conjugated linoleic acid have been reported to improve HFD-induced metabolic dysfunction through suppressing of hepatic inflammasome in mice and reversed metabolic dysfunction in offspring (6, 38, 39). Taken together, these results suggest that MA improved diet-induced lipid abnormality and metabolic syndrome partly through activation of NLRP3 inflammasome in mother rats and offspring.

## Conclusions

The present study indicated that MA supplementation during pregnancy and lactation alleviated HFD-induced maternal-fetal hepatic nitrogen radical production and increased antioxidative enzymes, attenuated lipid accumulation and NLRP3 inflammasome, and improved hepatic function, suggesting MA can be promising functional constituents to prevent diet-induced chronic disease and metabolic syndrome.

## Supplementary Material

Inclusion of microbe-derived antioxidant during pregnancy and lactation attenuates high-fat diet-induced hepatic oxidative stress, lipid disorders, and NLRP3 inflammasome in mother rats and offspring

## References

[1] Cannon MV, Buchner DA, Hester J, Miller H, Sehayek E, Nadeau JH, et al. Maternal nutrition induces pervasive gene expression changes but no detectable DNA methylation differences in the liver of adult offspring. PLoS One 2014; 9: e90335. doi: 10.1371/journal.pone.0090335. 24594983 PMC3940881

[2] Heerwagen MJ, Miller MR, Barbour LA, Friedman JE. Maternal obesity and fetal metabolic programming: a fertile epigenetic soil. Am J Physiol Regul Integr Comp Physiol 2010; 299: R711. doi: 10.1152/ajpregu.00310.2010. 20631295 PMC2944425

[3] Gregorio BM, Souza-Mello V, Carvalho JJ, Mandarim-De-Lacerda CA, Aguila MB. Maternal high-fat intake predisposes nonalcoholic fatty liver disease in C57BL/6 offspring. Am J Obstet Gynecol 2010; 203: 495.e491–8. doi: 10.1016/j.ajog.2010.06.042. 20822767

[4] Ashino NG, Saito KN, Souza FD, Nakutz FS, Roman EA, Velloso LA, et al. Maternal high-fat feeding through pregnancy and lactation predisposes mouse offspring to molecular insulin resistance and fatty liver. J Nutrl Biochem 2012; 23: 341–8. doi: 10.1016/j.jnutbio.2010.12.011. 21543214

[5] Benatti RO, Melo AM, Borges FO, Ignacio-Souza LM, Simino LA, Milanski M, et al. Maternal high-fat diet consumption modulates hepatic lipid metabolism and microRNA-122 (miR-122) and microRNA-370 (miR-370) expression in offspring. Br J Nutr 2014; 111: 2112–22. doi: 10.1017/S0007114514000579. 24666709

[6] Zhu MJ, Kang Y, Xue Y, Liang X, García M, Rodgers D, et al. Red raspberries suppress NLRP3 inflammasome and attenuate metabolic abnormalities in diet-induced obese mice. J Nutr Biochem 2017; 53: 96. doi: 10.1016/j.jnutbio.2017.10.012. 29202274

[7] Vandanmagsar B, Youm YH, Ravussin A, Galgani JE, Stadler K, Mynatt RL, et al. The NLRP3 inflammasome instigates obesity-induced inflammation and insulin resistance. Nat Med 2011; 17: 179–88. doi: 10.1038/nm.2279. 21217695 PMC3076025

[8] Wen H, Gris D, Lei Y, Jha S, Zhang L, Huang MT, et al. Fatty acid-induced NLRP3-PYCARD inflammasome activation interferes with insulin signaling. Nat Immunol 2011; 12: 408. doi: 10.1038/ni.2022. 21478880 PMC4090391

[9] Li J, Cordero P, Solanki A, Vinciguerra M, Crompton T, Oben JA. Maternal obesity programs offspring’s liver immune cells intra-uterine. J Hepatol 2018; 68: S353–4. doi: 10.1016/S0168-8278(18)30930-9.

[10] Li S, Tan H-Y, Wang N, Zhang Z-J, Lao L, Wong C-W, et al. The role of oxidative stress and antioxidants in liver diseases. Int J Mol Sci 2015; 16: 26087–124. doi: 10.3390/ijms161125942. 26540040 PMC4661801

[11] Blasa M, Angelino D, Gennari L, Ninfali P. The cellular antioxidant activity in red blood cells (CAA-RBC): a new approach to bioavailability and synergy of phytochemicals and botanical extracts. Food Chem 2011; 125: 685–91. doi: 10.1016/j.foodchem.2010.09.065.

[12] Xu J, Xu C, Chen X, Cai X, Yang S, Sheng Y, et al. Regulation of an antioxidant blend on intestinal redox status and major microbiota in early weaned piglets. Nutrition 2014; 30: 584–9. doi: 10.1016/j.nut.2013.10.018. 24698350

[13] Cai X, Chen X-L, Yang F, Xu J-X, Gu J, Zhang C. A preliminary research of antioxidant capacity by micro-derived antioxidants in vitro. Biotechnology 2011; 21: 84–7. doi: 10.3969/j.issn.1004-311X.2011.06.160

[14] Zhao S, Li S, Xu X, Zhong Q, Zhang J, Xu W, et al. Effects of micro-derived antioxidants on antioxidant capacity and litter size in high fat diet-induced pregnant rats. J. Sichuan Agricult Univ 2017; 35: 594–8. doi:10.16036/j.issn.1000-2650.2017.04.021

[15] Carmiel-Haggai M, Cederbaum AI, Nieto N. A high-fat diet leads to the progression of non-alcoholic fatty liver disease in obese rats. FASEB J 2005; 19: 136–8. doi: 10.1096/fj.04-2291fje. 15522905

[16] Dowman JK, Tomlinson JW, Newsome PN. Pathogenesis of non-alcoholic fatty liver disease. QJM Mon J Assoc Phys 2010; 103: 71. doi: 10.1093/qjmed/hcp158. PMC2810391 19914930

[17] Klop B, Elte JW, Cabezas MC. Dyslipidemia in obesity: mechanisms and potential targets. Nutrients 2013; 5: 1218–40. doi: 10.3390/nu5041218. 23584084 PMC3705344

[18] Yamaguchi R, Nakagawa Y, Liu YJ, Fujisawa Y, Sai S, Nagata E, et al. Effects of maternal high-fat diet on serum lipid concentration and expression of peroxisomal proliferator-activated receptors in the early life of rat offspring. Horm Metab Res 2010; 42: 821–5. doi: 10.1055/s-0030-1261954. 20711951

[19] Zhang X-L, Wu Y-F, Wang Y-S, Wang X-Z, Piao C-H, Liu J-M, et al. The protective effects of probiotic-fermented soymilk on high-fat diet-induced hyperlipidemia and liver injury. J Funct Foods 2017; 30: 220–7. doi: 10.1016/j.jff.2017.01.002.

[20] Nido SA, Shituleni SA, Mengistu BM, Liu Y, Khan AZ, Gan F, et al. Effects of selenium-enriched probiotics on lipid metabolism, antioxidative status, histopathological lesions, and related gene expression in mice fed a high-fat diet. Biol Trace Elem Res 2016; 171: 399–409. doi: 10.1007/s12011-015-0552-8. 26546553

[21] Choi S-B, Lew L-C, Yeo S-K, Nair Parvathy S, Liong M-T. Probiotics and the BSH-related cholesterol lowering mechanism: a Jekyll and Hyde scenario. Crit Rev Biotechnol 2015; 35: 392–401. doi: 10.3109/07388551.2014.889077. 24575869

[22] Jeun J, Kim S, Cho S-Y, Jun H-J, Park H-J, Seo J-G, et al. Hypocholesterolemic effects of Lactobacillus plantarum KCTC3928 by increased bile acid excretion in C57BL/6 mice. Nutrition 2010; 26: 321–30. doi: 10.1016/j.nut.2009.04.011. 19695834

[23] Bogdan C. Nitric oxide synthase in innate and adaptive immunity: an update. Trends Immunol 2015; 36: 161–78. doi: 10.1016/j.it.2015.01.003. 25687683

[24] Miyata T, Eckardt K, Nangaku M. Studies on renal disorders, oxidative stress in applied basic research and clinical practice, 2010. doi: 10.1007/978-1-60761-857-7.

[25] Schönfeld P, Wojtczak L. Fatty acids as modulators of the cellular production of reactive oxygen species. Free Radical Biol Med 2008; 45: 231–41.doi: 10.1016/j.freeradbiomed.2008.04.029. 18482593

[26] Chen P, Gu Y, Yu S, Xu J. Protective effects of microbe-derived antioxidant on lipopolysaccharide-induced liver injury in rats. J. Shanghai Jiao Tong Univ (Agri Sci), 2016; 34: 17–22. doi: 10.3969/J.ISSN.1671-9964.2016.05.003

[27] Chen H, Jiang Y, Yang Z, Hu W, Xiong L, Wang N, et al. Effects of Chimonanthus nitens Oliv. Leaf extract on glycolipid metabolism and antioxidant capacity in diabetic model mice. Oxid Med Cell Longev 2017; 2017: 7648505. doi: 10.1155/2017/7648505. 29057036 PMC5625751

[28] Ros P, Díaz F, Freire-Regatillo A, Argente-Arizón P, Barrios V, Argente J, et al. Resveratrol intake during pregnancy and lactation modulates the early metabolic effects of maternal nutrition differently in male and female offspring. Endocrinology 2017; 159: 810–25. doi: 10.1210/en.2017-00610. 29186387

[29] Mittal M, Chatterjee S, Flora SJS. Combination therapy with vitamin C and DMSA for arsenic–fluoride co-exposure in rats. Metallomics 2018; 10: 1291–306. doi: 10.1039/c8mt00192h. 30140832

[30] Luo Z, Zhu W, Guo Q, Luo W, Zhang J, Xu W, et al. Weaning induced hepatic oxidative stress, apoptosis, and aminotransferases through MAPK signaling pathways in piglets. Oxid Med Cell Longev 2016; 2016: 4768541. doi: 10.1155/2016/4768541. 27807471 PMC5078666

[31] Riedl SJ, Shi Y. Molecular mechanisms of caspase regulation during apoptosis. Nat Rev Mol Cell Biol 2004; 5: 897. doi: 10.1038/nrm1496. 15520809

[32] Hyder MA, Hasan M, Mohieldein AH. Comparative levels of ALT, AST, ALP and GGT in liver associated diseases. Eur J Exp Biol 2013; 3: 280–4. ISSN: 2248-9215CODEN (USA):EJEBAU

[33] Kim J, Kim J, Kwon YH. Effects of disturbed liver growth and oxidative stress of high-fat diet-fed dams on cholesterol metabolism in offspring mice. Nutr Res Pract 2016; 10: 386–92. doi: 10.4162/nrp.2017.11.5.435. 27478544 PMC4958640

[34] McCurdy CE, Bishop JM, Williams SM, Grayson BE, Smith MS, Friedman JE, et al. Maternal high-fat diet triggers lipotoxicity in the fetal livers of nonhuman primates. J Clin Invest 2009; 119: 323–35. doi: 10.1172/JCI32661. 19147984 PMC2631287

[35] Lv Z, Fan H, Zhang B, Ning C, Xing K, Guo Y. Dietary genistein supplementation in laying broiler breeder hens alters the development and metabolism of offspring embryos as revealed by hepatic transcriptome analysis. FASEB J 2018; 32: 4214–28. doi: 10.1096/fj.201701457R. 29518347

[36] Boden G. Interaction between free fatty acids and glucose metabolism. Curr Opin Nutr Metab Care 2002; 5: 545–9. doi: 10.1097/00075197-200209000-00014 12172479

[37] Legrand-Poels S, Esser N, L’homme L, Scheen A, Paquot N, Piette J. Free fatty acids as modulators of the NLRP3 inflammasome in obesity/type 2 diabetes. Biochem Pharmacol 2014; 92: 131–41. doi: 10.1016/j.bcp.2014.08.013. 25175736

[38] Porras D, Nistal E, Martínez-Flórez S, Pisonero-Vaquero S, Olcoz JL, Jover R, et al. Protective effect of quercetin on high-fat diet-induced non-alcoholic fatty liver disease in mice is mediated by modulating intestinal microbiota imbalance and related gut-liver axis activation. Free Radical Biol Med 2017; 102: 188–202. doi: 10.1016/j.freeradbiomed.2016.11.037. 27890642

[39] Segovia SA, Vickers MH, Zhang XD, Gray C, Reynolds CM. Maternal supplementation with conjugated linoleic acid in the setting of diet-induced obesity normalises the inflammatory phenotype in mothers and reverses metabolic dysfunction and impaired insulin sensitivity in offspring. J Nutr Biochem 2015; 26: 1448–57. doi: 10.1016/j.jnutbio.2015.07.013. 26318151

[40] Wang YL, Han QQ, Gong WQ, Pan DH, Wang LZ, Hu W, et al. Microglial activation mediates chronic mild stress-induced depressive- and anxiety-like behavior in adult rats. J Neuroinflamm 2018; 15: 21.doi: 10.1186/s12974-018-1054-3. PMC5773028 29343269

[41] Long F, Wang N, Zhang R, Qiu H, Lv J, Wang X, et al. Effects of transplantation of bone marrow mesenchymal stem cells on hepatic injury and metabolism in rats with acute liver failure. Int J Clin Exp Med 2017; 10: 4881–8. doi: 1940-5901/IJCEM0045622

[42] Zhu JL, Chen Y, Liu J. Effects of nonylphenol on prostate cell apoptosis and expressions of caspase-3,caspase-8,and caspase-9 mRNA in male Wistar rats. Chinese J Pub Health 2013; 29: 1187–9. doi: 10.11847/zgggws2013-29-08-29

[43] Ashok I, Wankhar D, Wankhar W, Sheeladevi R. Neurobehavioral changes and activation of neurodegenerative apoptosis on long-term consumption of aspartame in the rat brain. J Nutr Intermed Metab 2015; 2: 76–85. doi: 10.1016/j.jnim.2015.09.001.

[44] Huang Y, Ye T, Liu C, Fang F, Chen Y, Dong Y. Maternal high-fat diet during pregnancy and lactation affects hepatic lipid metabolism in early life of offspring rat. J Biosciences 2017; 42: 311–19. doi: 10.1007/s12038-017-9675-8. 28569254

[45] Tang B, Zhang JG, Tan HY, Wei XQ. Astragaloside IV inhibits ventricular remodeling and improves fatty acid utilization in rats with chronic heart failure. Bioscience Rep 2018; 38: BSR20171036. doi: 10.1042/BSR20171036. PMC6048210 29301869

[46] Ning J, Sun Q, Qian S, Dang S, Chen G. Taurine promotes cognitive function in prenatally stressed juvenile ratsviaactivating the Akt-CREB-PGC1α pathway. Redox Biol 2016; 10: 179–90. doi: 10.1016/j.redox.2016.10.004. 27768969 PMC5072153

