# Supplementary material for: Inclusion of microbe-derived antioxidant during pregnancy and lactation attenuates high-fat diet-induced hepatic oxidative stress, lipid disorders, and NLRP3 inflammasome in mother rats and offspring
Source: Food Nutr Res. 2019 Aug 23;63:10.29219/fnr.v63.3504. doi: 10.29219/fnr.v63.3504 (PMC8153845; doi:10.29219/fnr.v63.3504)
Supplement: Inclusion of microbe-derived antioxidant during pregnancy and lactation attenuates high-fat diet-induced hepatic oxidative stress, lipid disorders, and NLRP3 inflammasome in mother rats and offspring [file FNR-63-3504-s001.docx]

Supplemental Table 1 The liver weight of mother rats at L1 and L10

|  | CG | HFD | HFDA |
| --- | --- | --- | --- |
| Liver weights of mother rats |  |  |  |
| L1 (g) | 14.67 ± 0.87^a^ | 13.92 ± 1.34^a^ | 10.80 ± 1.81^b^ |
| L10 (g) | 11.58 ±1.35 | 11.15 ± 1.67 | 11.23 ± 0.72 |

Data were presented as mean ± standard deviation (SD). Values with different letters differ significantly (*p* < 0.05). CG: control group; HFD: high fat diet; HFDA: HFD + 2% microbe-derived antioxidant (n=6).
